# Supplementary material for: Large-Area Thermal Distribution Sensor Based on Multilayer Graphene Ink
Source: Sensors (Basel). 2020 Sep 11;20(18):5188. doi: 10.3390/s20185188 (PMC7570513; doi:10.3390/s20185188)
Supplement: Supplementary file 1 [file sensors-20-05188-s001.pdf]

Large-Area Thermal Distribution Sensor Based on Multilayer Graphene Ink

Tomi Koskinen *, Taneli Juntunen and Ilkka Tittonen

Department of Electronics and Nanoengineering, Aalto University, P.O. Box 13500, FI-00076 Aalto, Finland; taneli.juntunen@iki.fi (T.J.); ilkka.tittonen@aalto.fi (I.T.)

* Correspondence: tomi.koskinen@aalto.fi

1. Temperature-based water level detection

The temperature distribution sensing setups with the sensor attached onto the outer surface of a 1 L beaker with 400 mL and 600 mL water inside the beaker are presented in Figures S1 (a) and (b), respectively. The hot and cold experiments with temperatures 36 °C and 13 °C, along with corresponding temperature maps, are presented on the center and rightmost positions of each subfigure, respectively.

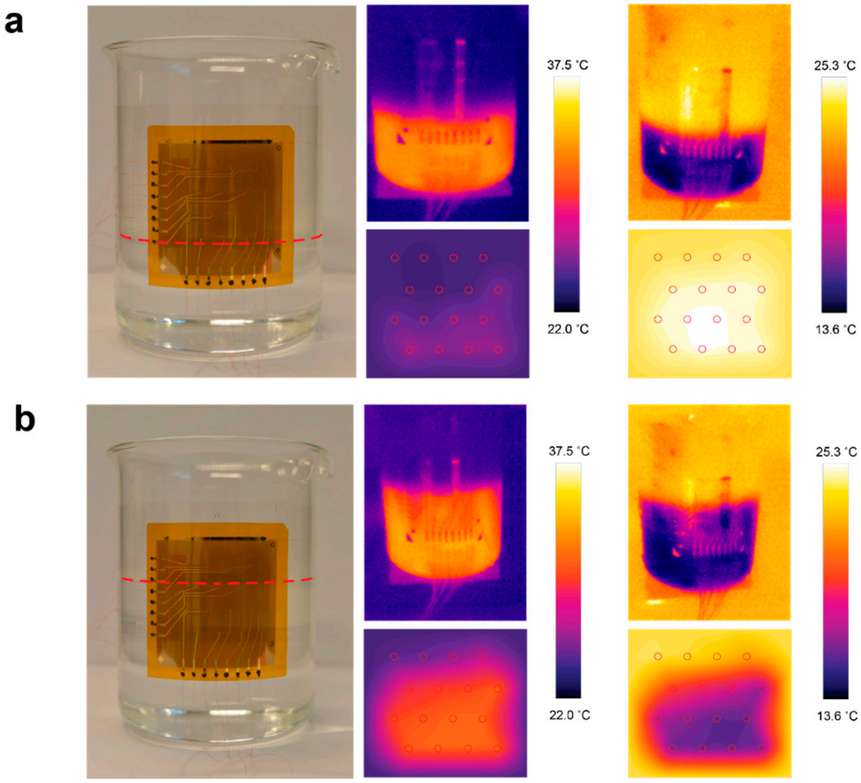

Figure S1. Water level detection. Sensor attached onto the outer surface of a 1L beaker with 400 mL (a) and 600 mL (b) water inside. The water temperatures corresponding to hot and cold images are 36 °C and 13 °C, respectively.

2. Film resistance under controlled deformation

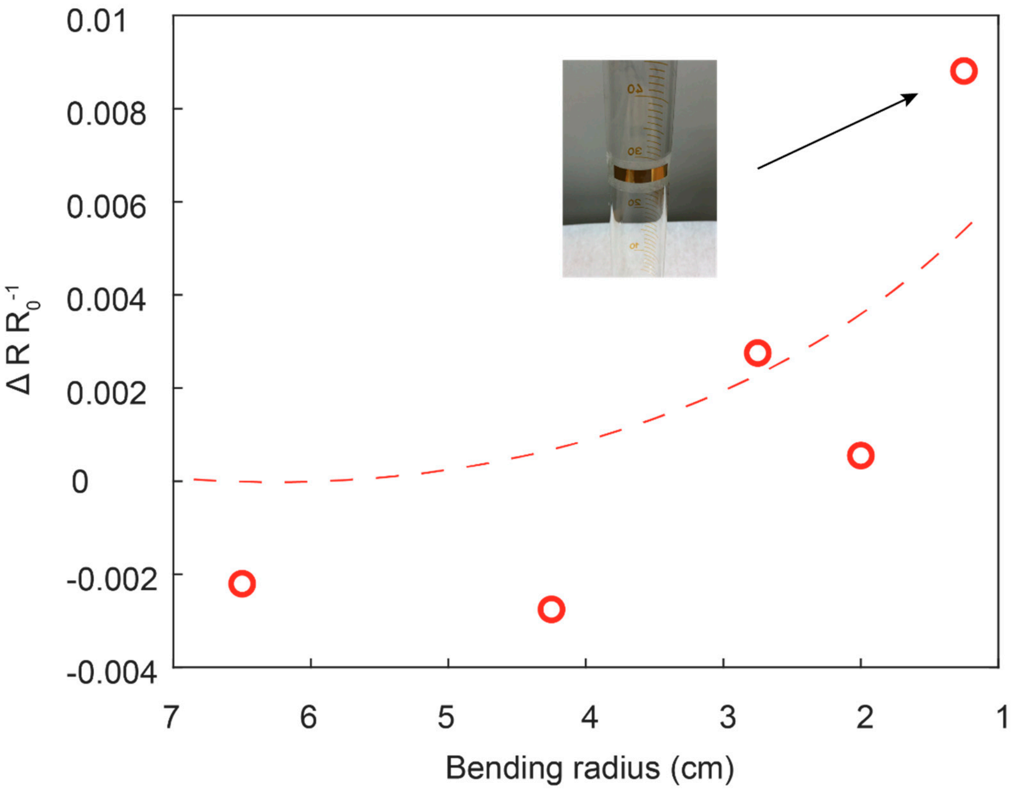

Figure S2. Relative change in multilayer graphene film resistance as a function of bending radius. The dashed line acts as a guide to the eye. The film resistance stays approximately unchanged until very small bending radii, implying that the electrical properties of the sensor are not affected by deformation in typical thermoelectric sensing applications.

3. Film resistance under controlled humidity

The effect of humidity on the resistance of the multilayer graphene film is presented in Figure S3. The sample was kept in a closed chamber with controlled humidity. The resistance shows an increasing trend with increasing relative humidity.

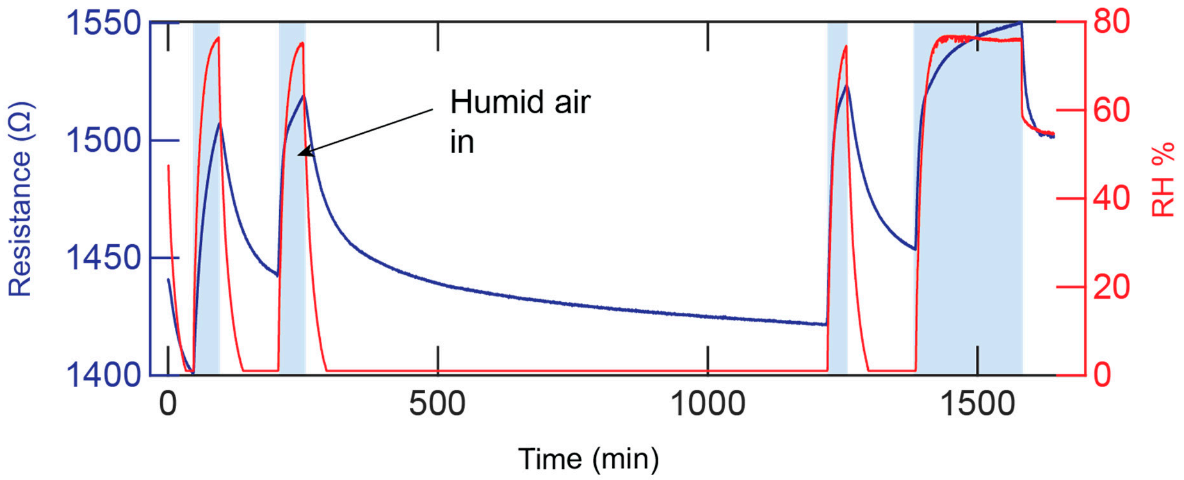

Figure S3. Resistance of a multilayer graphene film as a function of time when predisposed to different humidity conditions. The resistance increases with increasing humidity.

4. Error between different pixels at the same temperature

The signals produced during the water level detection experiment of Figure 3(a) by the pixels of the two bottom rows of the sensor are presented in Figure S4. Mean signal level at the steady state is 309 μV with a standard deviation of 15.8 μV. Translated to temperature via Seebeck coefficient S = 43.2 μV K^{-1}, the standard deviation is 0.36 K. Therefore, the error between different legs stays relatively small and the signals have a high uniformity.

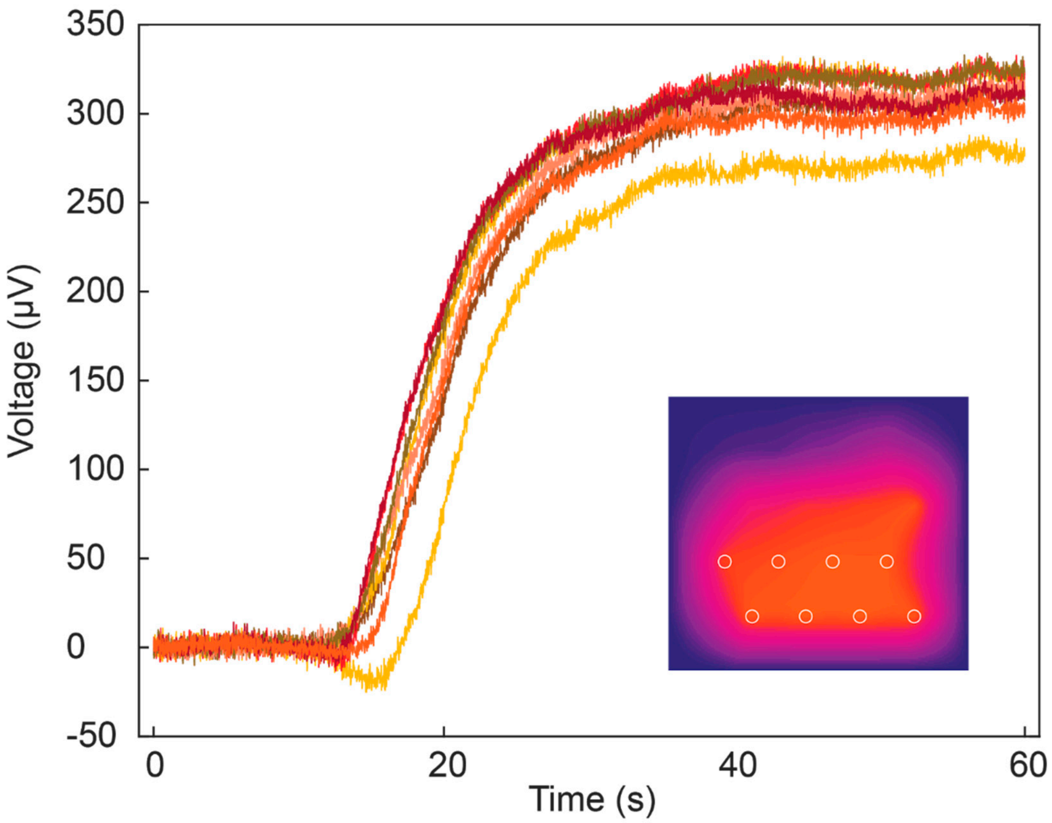

Figure S4. Voltage signals from the pixels of the two bottom rows in the water level sensing experiment of Figure 3 (a). The inset shows the pixel positions and the resulting temperature distribution map. .

5. Finite-element method model

Figure S5 (a) presents the finite-element mesh used in the COMSOL FEM simulations. Figure S5 (b) presents the temperature distribution in the domain immediately after shutting off the boundary heat source. The heat source is centered on top of a boundary probe representing a pixel in the sensor structure. The heat pulse is of form

$$p(x_1, x_2, x_3) = p_{finger}\left(T_{finger} - T(x_1, x_2, x_3)\right)\prod_{i=1}^{3}\frac{1}{\sigma_i\sqrt{2\pi}}exp(-\frac{(x_i - x_{i,0})^2}{2{\sigma_i}^2}),$$

where x_1 and x_2 are spatial coordinates and x_3 is the temporal dimension. p_{finger} is the heating power, σ_i is the spot size, while T and T_{finger} are temperature and maximum temperature of the heating [S1].

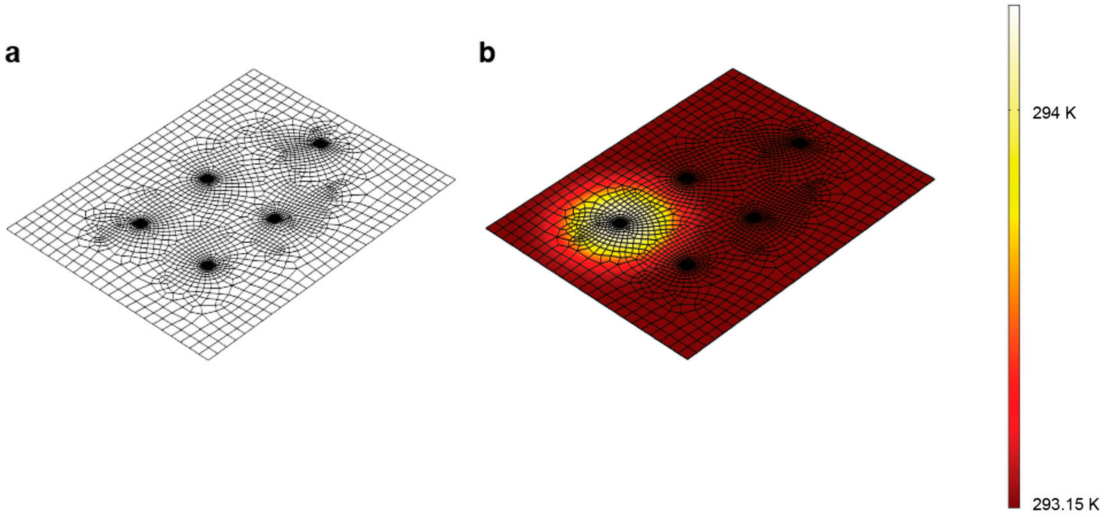

Figure S5. FEM model of the sensor. (a), meshed domain showing four readout probes and a reference ground. (b), heat pulse introduced on the surface, positioned on top of a voltage probe. The color bar represents the temperature of the domain.

Parameters for domain geometry and relevant thermophysical constituents are included in Table S1 below.

Table S1. FEM parameters used in the simulations.

**Geometry parameters**	**Value**
Domain x dimension	0.03 m
Domain y dimension	0.0375 m
Substrate thickness	50 μm
Thermoelectric film thickness	100 nm
Boundary probe diameter	1 mm
**Thermoelectric parameters**	
Thermal conductivity	1 W m^{-1} K^{-1}
Electrical conductivity	12500 S m^{-1}
Heat capacity	720 J kg^{-1} K^{-1}
Seebeck coefficient	43.2 μV K^{-1}
Density	500 kg m^{-3}
Substrate thermal conductivity	0.12 W m^{-1} K^{-1}
**Other**	
Heating power	50 W
Ambient temperature	293.15 K
Cutoff temperature	1.2 K
Heating spot diameter	6 mm
Heat transfer coefficient	5 W m^{-1} K^{-1}

[S1] M. Ruoho, T. Juntunen, T. Alasaarela, M. Pudas, I. Tittonen, Adv. Mater. Technol. 2016, 1, 1600204.
